# The Effect of Sleeve Gastrectomy on Pregnancy Complications: A Cross-Sectional Study in Saudi Arabia

**DOI:** 10.7759/cureus.40157

**Published:** 2023-06-08

**Authors:** Ghaida A. Eissa, Rahaf A Khurmi, Teaf J Holbah, Durrah W Alabdullah, Sarah A Aleban, Ajyal A Aljohani, Saleha M Zaidan, Ahlam M Hakami

**Affiliations:** 1 Faculty of Medicine, King Abdulaziz University, Jeddah, SAU; 2 Faculty of Medicine, Taibah University, Medina, SAU; 3 Faculty of Medicine, Jazan University, Jazan, SAU; 4 Faculty of Medicine, Princess Nourah Bint Abdulrahman University, Riyadh, SAU; 5 Obstetrics and Gynecology, Jazan University, Jazan, SAU

**Keywords:** bariatric surgery, obesity related complications, obesity, maternal-fetal medicine, gastric sleeve surgery

## Abstract

Background: Obesity is defined as abnormal or excessive fat accumulation that may impair health. Until recently, the only effective method for treating morbid obesity over the long term was bariatric surgery (BS). During pregnancy, obesity is correlated with higher risks for numerous complications, including gestational diabetes mellitus, pre-eclampsia, mortality, and large-for-gestational-age neonates. The most commonly reported complications among women who underwent sleeve gastrectomy and experienced pregnancy were placental bleeding, oligohydramnios, urinary tract infection, appendicitis, and recurrent abortions.

Objectives: We aim to estimate the consequence of sleeve gastrectomy and its relation with pregnancy outcomes among women in Saudi Arabia.

Methodology: This study adopted a quantitative, descriptive, cross-sectional design. It was conducted in Saudi Arabia between February and May 2023 among women who became pregnant after undergoing sleeve gastrectomy.

Result: Anemia was experienced by 78.8% of the patients during pregnancy. In our study, 18% of the individuals experienced complications during or right after delivery, with postpartum hemorrhage being the most frequent (43.1%). We discovered that pre-eclampsia and delivering a baby small for gestational age were considerably more common in pregnant women who smoked (p ≤ 0.05). On the other hand, no significant association was discovered between any comorbidity and mode of delivery, birth weight, child complications, or difficulties that occurred during or right after labor.

Conclusion: We concluded that weight gain after sleeve gastrectomy negatively impacted pregnancy and increased the probability of several complications for the mother and fetus. Healthcare providers must inform every woman undergoing BS about the possible complication of an unhealthy lifestyle after the procedure.

## Introduction

Obesity is defined as abnormal or excessive fat accumulation that may impair health; it is currently considered an epidemic. It is still a substantial health issue worldwide, and the number of affected individuals is rising [1,2]. Weight loss procedures have become the most effective treatment for morbid obesity [1]. The rate of obesity among females is greater than that of males in Saudi society, where it is 33.5% opposed to 24.1% [3]. Until now, surgical intervention, “Bariatric surgery (BS)”, is the only practical approach for the long‐term treatment of morbid obesity [1,2]. Today, Roux-en-Y gastric bypass, sleeve gastrectomy (SG), and adjustable gastric banding are the most popular and commonly performed bariatric surgeries (BSs) [4]. Eighty percent of patients who undergo BSs are women of childbearing age [5]. With obesity, other complications such as type 2 diabetes mellitus (DM), hypertension (HTN), and hyperlipidemia occur. Still, obesity in women of childbearing age poses added complications such as polycystic ovarian syndrome and infertility [6,7].

Moreover, fertility in women with obesity generally improves after BS, as ovulatory problems and menstrual irregularities often resolve after weight loss [8,9]. During pregnancy, obesity is correlated with higher risks for numerous complications, including gestational diabetes mellitus (GDM) [10], pregnancy-associated HTN and pre-eclampsia [11,12], preterm birth [13], failure to progress in labor [14], a cesarean delivery [15], complications related to macrosomia, venous thromboembolism [16], infection [17], depression [18], congenital anomalies [19], mortality [20], prematurity and large for gestational age (LGA) neonates [21]. In addition, studies show the benefits of a BS in reducing the complications related to obesity that may affect pregnancy and neonatal outcomes [22,23]. 

A recent study on neonatal outcomes following maternal BS reported that infants who were small for gestational age (SGA) were more common among mothers with insufficient weight gain. Conversely, LGA infants were more common among mothers who had excessive weight gain when compared to the other gestational weight gain groups. The insufficient-weight-gain group also had a greater preterm birth rate [24]. According to a study published in 2018, the most commonly reported complications among women who underwent SG and experienced pregnancy were placental bleeding, oligohydramnios, urinary tract infection (UTI), appendicitis, and recurrent abortions [25]. In contrast, a study conducted by Wang et al., who studied the pregnancy outcomes after BSs, documented that none of the patients had delivery-related complications. The growth and development of the newborn have been standard since the birth follow-up [26].

However, no previous study was conducted in Saudi Arabia to assess the impact of SG on pregnancy outcomes. The aim of this study is to estimate the consequence of SG and its relation with pregnancy outcomes in women in Saudi Arabia. Also, as a secondary aim, we explore the possible complications that develop for the mother and the fetus after gaining weight following BS procedures.

## Materials and methods

Study design and setting

This study adopted a quantitative, descriptive, cross-sectional design. It was conducted in Saudi Arabia between February 2023 and May 2023 among women who became pregnant after undergoing SG. Women who became pregnant more than twice after the procedure and refused to participate in the survey were ruled out. This study's primary goal is to determine SG's impact and its relationship with pregnancy outcomes among women of childbearing age living in Saudi Arabia.

Sample size and sampling procedure

The sample size calculated for this study was 245 participants. The selection was made using a convenience sampling technique, with a 95% confidence level and a 5% margin of error. The calculations were made using a Raosoft sample size calculator [27].

Data collection instrument

An online survey obtained the information, and the participants' responses were securely entered into an electronic Google Forms questionnaire. The questionnaire was divided into three sections. Section 1 covered the respondents' sociodemographic data, which included age, nationality, region of residence, educational level, income, occupational status, smoking exposure, current medical status, height, and weight. Section 2 included a detailed pregnancy history following the procedure, beginning with three questions about body weight at different events, possible pregnancy complications, mode of delivery (MOD), and type of delivery. Finally, a "Yes" or "No" question about maternal complications in the delivery, such as vaginal bleeding, infection, placenta accreta, and placenta previa. The last segment of the survey involved evaluating the newborn's outcomes by inquiring about the newborn's weight and comorbidities (structural abnormalities, intrauterine growth restriction, dead fetus, and neonatal intensive care unit [NICU] admission).

Data analysis

Data were analyzed using the SPSS program version 26 (IBM Corp, Armonk, NY). To investigate the association between the variables, the chi-squared (χ^2^) test was applied to qualitative data that were expressed as numbers and percentages. The association between the quantitative non-parametric variables that were expressed as mean and standard deviation (mean ± SD) was examined using the Mann-Whitney test. Correlation analysis was performed using Spearman's test and statistical significance was defined as a p-value of less than 0.05.

Research ethics

This cross-sectional study was authorized by The Standing Committee for Scientific Research at Jazan University in Jazan, Kingdom of Saudi Arabia. The questionnaire's anonymity was chosen to maintain the privacy of the participant's responses.

## Results

This study involved 245 participants. The participant's demographic data are shown in Table 1. We demonstrate that 4.9% of studied females were smoking during pregnancy. Of them, 20% had chronic diseases, with DM (38.7%) and HTN (34.6%) being the most common. The mean weight at the beginning of pregnancy was 46.52 ± 16.01 kg, the mean weight gain during pregnancy was 15.62 ± 16.48 kg, and the mean weight last measured before birth was 74 ± 15.731 kg. Of the participants, 78.8% had anemia during pregnancy.

**Table 1:** Distribution of studied females according to their demographics and BMI

Variable	No. (%)
Age (years)	
<30	94 (38.4)
30-39	101 (41.2)
40-49	43 (17.6)
>50	7 (2.9)
Nationality	
Saudi	232 (94.7)
Non-Saudi	13 (5.3)
KSA region	
Southern	28 (11.4)
Eastern	16 (6.5)
Northern	50 (20.4)
Western	92 (37.6)
Central	59 (24.1)
Educational level	
Primary	7 (2.9)
Middle	5 (2)
Secondary	46 (18.8)
University	167 (68.2)
Postgraduate	20 (8.2)
Occupation	
Unemployed	121 (49.4)
Employed	124 (50.6)
BMI categories	
Underweight	6 (2.4)
Normal weight	94 (38.4)
Overweight	91 (37.1)
Obese	54 (22)
BMI (mean ± SD)	26.54 ± 4.85

**Figure 1:**
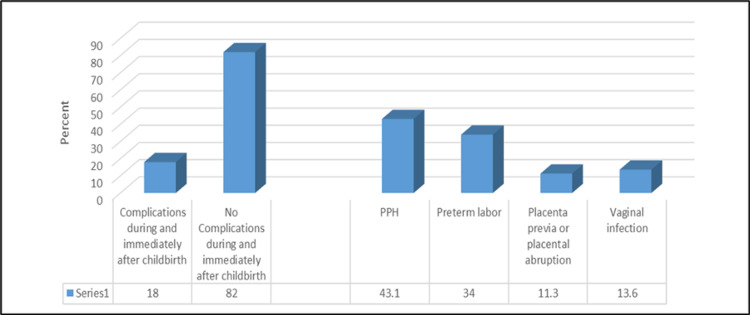
Percentage distribution of studied females according to prevalence and types of complications that happened during and immediately after childbirth PPH, postpartum hemorrhage.

**Figure 2:**
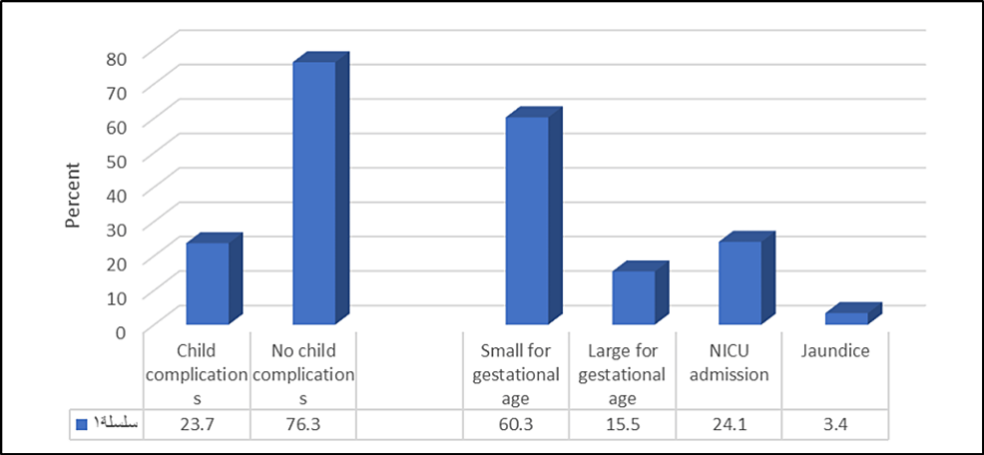
Percentage distribution of studied females according to prevalence and types of child complications NICU, neonatal intensive care unit.

Additionally, our study shows that 78% of females had a normal non-induced type of delivery, and 58% had a vaginal birth. Of them, 18% had complications during and immediately after childbirth, with postpartum hemorrhage (PPH) being the most common (43.1%). The mean baby weight at birth was 3.08 ± 1.42 kg, and 23.7% of babies had complications with being SGA as the most common complication (60.3%), as shown in Figures 1, 2.

As for the relationship between smoking status during pregnancy and other factors, Table 2 shows that females smoking during pregnancy had a significantly higher percentage of having pre-eclampsia, having a child with complications, and having a baby SGA (p ≤ 0.05). On the other hand, a non-significant relationship was found between having any comorbidity and pregnancy disorders, type of delivery and MOD, complications happening during and immediately after childbirth, and birth weight or child complications (p ≥ 0.05), as shown in Table 3. 

**Table 2:** Relationship between smoking status during pregnancy and pregnancy disorders, type and MOD, complications that happened during and immediately after childbirth, birth weight and child complications GDM, gestational diabetes mellitus; PPH, postpartum hemorrhage; NICU, neonatal intensive care unit. *Mann-Whitney test.

Variable	Smoking during pregnancy		χ^2^	p-Value
	No, no. (%)	Yes, no. (%)		
Disorders during pregnancy			1.54	0.213
No	157 (67.4)	6 (50)		
Yes	82 (32.6)	6 (50)		
If yes, specify: (No.: 82)				
Anemia	59 (25.3)	4 (33.3)	0.38	0.536
Pre-eclampsia	8 (3.4)	2 (16.7)	5.1	0.024
GDM	15 (6.4)	0 (0.0)	0.82	0.364
Type of delivery			1.38	0.24
Normal	180 (77.3)	11 (91.7)		
Induced	53 (22.7)	1 (8.3)		
Mode of delivery			0.43	0.932
Emergency cesarean section	20 (8.6)	1 (8.3)		
Scheduled cesarean section	52 (22.3)	2 (16.7)		
Vaginal birth	134 (57.5)	8 (66.7)		
Vaginal birth using auxiliary tools such as forceps	27 (11.6)	1 (8.3)		
Complications during and immediately after childbirth			0.42	0.515
No	192 (82.4)	9 (75)		
Yes	44 (17.6)	3 (25)		
If yes, specify: (No.:44)				
PPH	17 (7.3)	2 (16.7)	1.4	0.237
Preterm labor	14 (6)	1 (8.3)	0.1	0.743
Placenta previa or placental abruption	5 (2.1)	0 (0.0)	0.26	0.608
Postpartum vaginal infection	6 (2.6)	0 (0.0)	0.31	0.574
Has the child suffered any of the following complications			4.84	0.028
No	181 (77.7)	6 (50)		
Yes	52 (22.3)	6 (50)		
If yes, specify: (No.:52)				
Small for gestational age	29 (12.4)	6 (50)	13.14	<0.001
Large for gestational age	9 (3.9)	0 (0.0)	0.48	0.488
NICU admission	13 (5.6)	1 (8.3)	0.16	0.689
Jaundice	2 (0.9)	0 (0.0)	0.1	0.747
Baby weight at birth	3.09 ± 1.43	2.87 ± 1.36	0.008*	0.993

**Table 3:** Relationship between having any comorbidity and pregnancy disorders, type and MOD, complications happened during and immediately after childbirth, birth weight and child complications GDM, gestational diabetes mellitus; PPH, postpartum hemorrhage; NICU, neonatal intensive care unit; MOD, mode of delivery. *Mann-Whitney test.

Variable	Comorbidity		χ^2^	p-Value
	No, no. (%)	Yes, no. (%)		
Disorders during pregnancy			0.77	0.379
No	30 (61.2)	133 (67.9)		
Yes	19 (38.8)	63 (32.1)		
If yes, specify: (No.: 19)				
Anemia	14 (28.6)	49 (25)	0.26	0.609
Pre-eclampsia	5 (10.2)	5 (2.6)	5.86	0.015
GDM	0 (0.0)	15 (7.7)	3.99	0.046
Type of delivery			5.7	0.017
Normal	32 (65.3)	159 (81.1)		
Induced	17 (34.7)	37 (18.9)		
MOD			2.41	0.491
Emergency cesarean section	6 (12.2)	15 (7.7)		
Scheduled cesarean section	13 (26.5)	41 (20.9)		
Vaginal birth	24 (49)	118 (60.2)		
Vaginal birth using auxiliary tools such as forceps	6 (12.2)	22 (11.2)		
Complications during and immediately after childbirth			1.77	0.183
No	37 (75.5)	164 (83.7)		
Yes	12 (24.5)	32 (16.3)		
If yes, specify: (No.:12)				
PPH	7 (14.3)	12 (6.1)	3.65	0.056
Preterm labor	5 (10.2)	10 (5.1)	1.77	0.183
Has the child suffered any of the following complications			2.73	0.098
No	33 (67.3)	154 (78.6)		
Yes	16 (32.7)	42 (21.4)		
If yes, specify: (No.:16)				
Small for gestational age	9 (18.4)	26 (13.3)	0.83	0.361
Large for gestational age	5 (10.2)	4 (2)	7.38	0.007
NICU admission	2 (6.1)	11 (5.6)	0.01	0.891
Jaundice	0 (0.0)	2 (1)	0.5	0.478
Baby weight at birth	3.12 ± 1.52	2.89 ± 0.89	0.13*	0.891

As shown in Table 4, HTN participants had a significantly higher percentage of those who suffered from pregnancy disorders (p ≤ 0.05). At the same time, participants who had DM had a substantially higher rate of having a child who sustained any complications (p ≤ 0.05).

**Table 4:** Relationship between having HTN or DM as a comorbidity and pregnancy disorders and child complications HTN, hypertension; DM, diabetes mellitus.

Variable	HTN		χ^2^	p-Value
	No, no. (%)	Yes, no. (%)		
Disorders during pregnancy			8	0.005
No	157 (68.9)	6 (35.3)		
Yes	71 (31.1)	11 (64.7)		
Variable	DM			
	No, no. (%)	Yes, no. (%)		
Has the child suffered any of the following complications			3.87	0.049
No	176 (77.9)	11 (57.9)		
Yes	50 (22.1)	8 (42.1)		

## Discussion

This study aimed to estimate the impact of SG and its relation with pregnancy outcomes. Our results showed that the mean BMI of our participants was 26.54 ± 4.85 kg/m^2^, and 22% were obese. Twenty percent of our participants had chronic diseases, most of whom were diagnosed with DM (38.7%), and this significant percentage could be related to obesity [28]. We found that anemia is the most common disorder during pregnancy (76.8%). These results were consistent with those published by Wang X et al., which stated that anemia is the most common disorder among pregnant women [26]. Based on a recent systematic review, anemia is highly expected to occur after BS, with a prevalence of 17-77% of pregnancies [29]. However, since plasma volume expands faster than red cell mass throughout pregnancy, the high prevalence of anemia may be explained by the fact that hemoglobin concentration is anticipated to be at its lowest month by month [30]. Accordingly, to prevent and manage anemia during pregnancy, the guidelines suggest that nutritional supplementation be optimized three to six months before conception and that iron, ferritin, and transferrin levels be frequently checked [26]. GDM refers to DM that is first identified during the second or third trimester of pregnancy and is not immediately identifiable as type 1 or type 2 DM [31]. Furthermore, 33.5% of our participants had disorders during pregnancy, and 18.2% were diagnosed with GDM. Further, within one or two decades, the prevalence of GDM has been raised to be one of the most prevalent pregnancy problems. It has increased by more than 30% in many nations, especially developing countries [32]. A clear explanation of this considerable number is that recent research has observed that obesity increases the risk of developing GDM [31].

Our results were similar to a study done at King Khalid University Hospital and a specialized medical center which found that the majority of their participants had a spontaneous vaginal delivery [25]. Unexpectedly, 18% of our participants had complications compared to less than 5% in their study [25]. Although, recent studies have shown that BSs can increase the chance of adverse pregnancy outcomes [31].

Regarding the complications, we found that the most common complication after birth was PPH. A previous systematic review claims that the risk of PPH increases with increased BMI [33]. About one-fourth of the newborns in our study were SGA. These results were in line with a previous Saudi survey [25]. Our study showed that smoking is significantly associated with pregnancy-related complications, such as pre-eclampsia and having an SGA infant. Surprisingly, an earlier investigation by Wei J et al. stated that smoking during pregnancy has been shown to provide potential health benefits, including reducing the risk of pre-eclampsia [34].

On the other hand, our results showed a non-significant relationship between having any comorbidity and MOD, which is an alternative to a study published in Bahrain which showed that the rate of elective CS increased from 12.5% in non-diabetic mothers to 50% in pregnant women with pre-existing DM [35]. Additionally, a non-significant relationship was found between comorbidity and complications during and immediately after childbirth. An opposite result was obtained by Fakhraei et al., which concluded that women with a history of BS (particularly malabsorptive procedures), regardless of their current weight, benefit from care in a multidisciplinary team that includes Maternal Fetal Medicine due to increased risks of fetal growth restriction, preterm birth, and perinatal death [36]. Likewise, a non-significant relationship was found between having any comorbidity and birth weight or child complications which was differently stated in another previous study which showed that LGA newborns were demonstrated to be more common in pregnancies of women who lived with obesity and resulted in increased risks of cesarean section, birth injury, and admission to the NICU [36].

## Conclusions

This study concluded that weight gain after SG negatively impacted pregnancy and increased the probability of several complications for the mother and fetus, particularly anemia, GDM, and PPH. Women must be aware of these preventable harmful events. Furthermore, we reported that smoking is significantly associated with pregnancy-related complications among women who underwent BS, such as pre-eclampsia and having an SGA infant, a modifiable risk factor the mother should stop during pregnancy. Healthcare providers must inform every woman undergoing BS about the possible complication of an unhealthy lifestyle after the procedure that may affect her and her infant.
